# EEG Theta Dynamics within Frontal and Parietal Cortices for Error Processing during Reaching Movements in a Prism Adaptation Study Altering Visuo-Motor Predictive Planning

**DOI:** 10.1371/journal.pone.0150265

**Published:** 2016-03-10

**Authors:** Pieranna Arrighi, Luca Bonfiglio, Fabrizio Minichilli, Nicoletta Cantore, Maria Chiara Carboncini, Emily Piccotti, Bruno Rossi, Paolo Andre

**Affiliations:** 1 Neurorehabilitation Unit, University of Pisa, Pisa, Italy; 2 Department of Medicine Surgery and Neuroscience, University of Siena, Siena, Italy; 3 Unit of Environmental Epidemiology, Institute of Clinical Physiology, National Council of Research, Pisa, Italy; Universita degli Studi di Verona, ITALY

## Abstract

Modulation of frontal midline theta (fmθ) is observed during error commission, but little is known about the role of theta oscillations in correcting motor behaviours. We investigate EEG activity of healthy partipants executing a reaching task under variable degrees of prism-induced visuo-motor distortion and visual occlusion of the initial arm trajectory. This task introduces directional errors of different magnitudes. The discrepancy between predicted and actual movement directions (i.e. the error), at the time when visual feedback (hand appearance) became available, elicits a signal that triggers on-line movement correction. Analysis were performed on 25 EEG channels. For each participant, the median value of the angular error of all reaching trials was used to partition the EEG epochs into high- and low-error conditions. We computed event-related spectral perturbations (ERSP) time-locked either to visual feedback or to the onset of movement correction. ERSP time-locked to the onset of visual feedback showed that fmθ increased in the high- but not in the low-error condition with an approximate time lag of 200 ms. Moreover, when single epochs were sorted by the degree of motor error, fmθ started to increase when a certain level of error was exceeded and, then, scaled with error magnitude. When ERSP were time-locked to the onset of movement correction, the fmθ increase anticipated this event with an approximate time lead of 50 ms. During successive trials, an error reduction was observed which was associated with indices of adaptations (i.e., aftereffects) suggesting the need to explore if theta oscillations may facilitate learning. To our knowledge this is the first study where the EEG signal recorded during reaching movements was time-locked to the onset of the error visual feedback. This allowed us to conclude that theta oscillations putatively generated by anterior cingulate cortex activation are implicated in error processing in semi-naturalistic motor behaviours.

## Introduction

It is generally assumed that error signals play a role in shaping motor behaviour. When movements are planned the brain generates a prediction of the upcoming sensory feedback based on the current state and the efferent copy of the motor command [1]. If a movement has an unexpected sensory outcome, the brain generates an error signal. This error signal is used by the brain to adjust in real time motor commands, for example, to correct the ongoing hand trajectory in order to reach the goal [2]. In addition, the error signal is assumed to promote motor learning by training the brain to produce the optimum motor command (see [3] for ref.). Studies employing an alteration of the visual feedback (e.g., by applying prism or computer visual rotation) to induce execution errors indicate that both parietal and frontal structures participate in error elaboration, although differently [4]. Frontal regions may be essential for organizing on-line corrections in response to an unexpected trajectory error: in particular, they may become activated in novel situations, for example when a novel visuomotor rotation is introduced [4]. Parietal regions, instead, may cover the process of learning from errors introduced by the novel visuomotor rotation [4]. In particular, they may cover the process of learning and/or storing new visuomotor associations leading to error reduction through adaptation [5]. Moreover, once learning has occurred, parietal regions may also store the set of online corrections required to overcome the target displacement during the reach (the so called ‘target jump’) [6–8].

In a recent review, we suggested a scheme for movement correction that takes into account the magnitude of the prediction error in determining the mechanism of correction [9]. Our hypothesis was based on a known model which implies that, when an action is executed, sensory signals related to the movement reach the brain and are compared with the predicted sensory outcome (see [2] for ref.). In particular, we proposed that, if these sensory signals match fairly well the predicted outcome, the movement can be corrected automatically by the changes induced in the cortical areas involved in implicit automatic perceptual motor processes, such as the posterior parietal lobe and the premotor cortex (see [9] for ref.). By contrast, if the mismatch between the prediction and the evolving outcome is above the threshold for being automatically corrected, there is a further engagement of cortical areas, in particular the frontal ones, which are involved in the executive control of action. In support of these considerations, when tasks are novel (i.e., requiring high levels of attention) brain activation related to the task is widely distributed, but when a task becomes automatic activation is more localized and spreads more posteriorly (see [9] for ref.). There is evidence, in the literature pertaining to the cognitive domain, that medial prefrontal cortices (mPFC), including the anterior cingulate cortex (ACC), are key nodes of the network controlling executive functions and that frontal midline theta (fmθ) reflects information processing within them. In particular, ACC activation and related fmθ generation [10–12] have been involved in processing conflicting information in sensory streams [13], selection between alternative responses [14] and error detection [15]. Although an fmθ increase may follow both correct and incorrect responses in highly demanding tasks, fmθ increase is a far more robust phenomenon during error commission [16–19]. Moreover, error-related potentials (ERPs), such as the error-related negativity (ERN), which were considered to reflect the processing of an incorrect/conflicting response, may be generated from phase-resetting/-locking of ongoing theta activity in the mPFC/ACC [17–19]. These findings have led to the development of a model according to which ACC activation occurs whenever an action monitoring event is required [20–22]. Such an activation works to optimize goal-driven performance by favoring information gathering related to contextual cues or ongoing behaviours in order to implement the appropriate action selection/inhibition [20–22]. In the light of the evidence outlined above and in analogy with what happens in cognitive tasks, we suggest that mPFC, including the ACC, become also activated with motor error. Moreover, we suggest that theta oscillations may be the electrophysiological sign of that activation.

Unfortunately, we know very little about the role that the fronto-central theta band plays in regulating action correction following motor errors. In fact, while error monitoring during conflict/interference tasks has been largely investigated [23–25] in the cognitive domain, conversely, changes of fmθ and ERP amplitude in relation to errors during complex motor behaviour in semi-realistic settings have received little attention. In two visuo-motor tracking studies -requiring participants to keep a cursor within a visual zone [26] or to follow a sinusoidally moving visual trace [27]- theta oscillations associated with error execution phasically increased in the supplementary motor area [26] and the ACC [27], respectively. During movements under distorted visual feedback of the virtual hand, Contreras-Vidal and Kerick [28] identified a transient post-movement theta activity preceded by a fronto-central negative peak early after movement onset. Finally, recent studies on ERPs, presumably generated in the medial frontal cortex, found that the amplitude of the evoked responses following an error were parametrically modulated by the size of the hand path deviation induced either by a visual shift [29–30] or by a sudden variation of a force field [31]. In most of the aforementioned studies theta oscillations or evoked potentials were not locked to any anchor time point which was endowed with a meaning in the motor error domain, such as the appearance of visual feedback or the beginning of corrective movements. The only exception was the study of Anguera et al. [29] which time-locked their signals to the onset of online movement corrections. This study, however, did not specifically focus on theta oscillations, but rather on ERPs. Most importantly, none of the studies quoted above time-locked the EEG signals to the precise time point of the error visual feedback. Therefore, we designed a task in which the dependence of theta oscillations on the magnitude of the error visual feedback could be studied at the precise time of its appearance. At this aim, individual reaching movements were performed under partial visual occlusion of the arm and in the presence of a variable degree of visual shift induced by prisms. We postulate that spatial discrepancy between predicted and actual directions of movement at the time when visual feedback appears, elicits an error signal that varies according to the degree of discrepancy and in turn triggers movement correction. We used the instants when significant kinematically-identified events (i.e., feedback appearance or onset of correction) occur as suitable anchor points for analysis of error-related EEG spectral changes. In other words, this experimental design allowed us to study the relationship between theta activation and the magnitude of error when participants detected and/or corrected their kinematic errors. More precisely, in this experimental setting we test the following hypotheses: 1) theta activity (putatively originated in the mPFC/ACC) is scaled by the magnitude of the error; and 2) a temporal link exists between these brain dynamics and relevant kinematics events of movement corrections.

## Materials and Methods

### Participants

Twelve healthy male University students (age, 23.8 ± 2.8 yrs, mean ± S.D., all right-handed) took part in the study. They reported no history of neurological or psychiatric disease, no current psychoactive medication use and normal or corrected-to-normal vision. The study was conducted in accordance with the indications of the Declaration of Helsinki and was approved by the local ethics committee (Comitato Etico Sperimentazione del Farmaco, Azienda Ospedaliero-Universitaria Pisana). Written informed consent for all the healthy volunteers was obtained from all participants.

### Experimental apparatus

**Fig 1:**
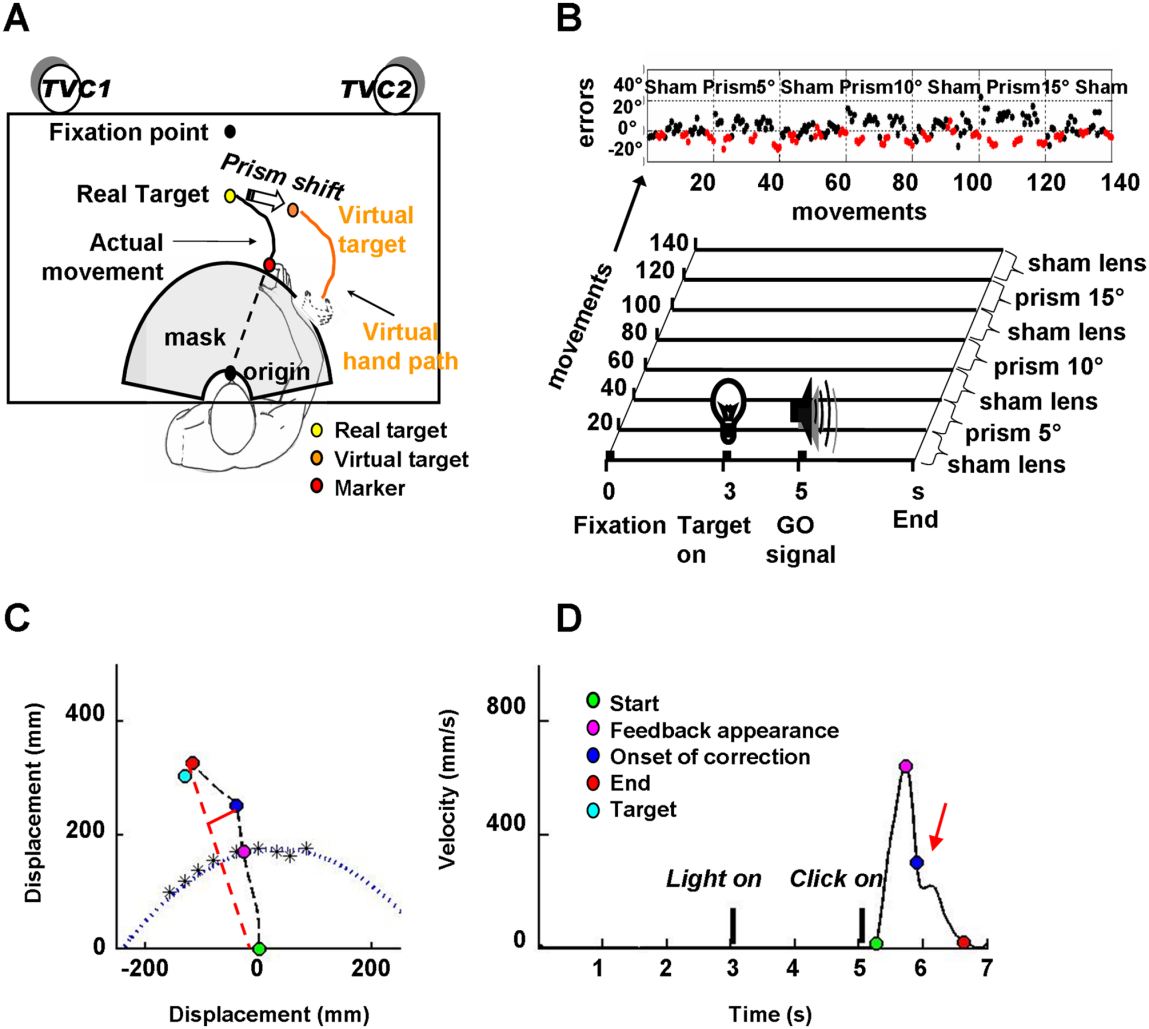
Experimental set up and protocol. A. Experimental set up. For sake of simplicity only the target at 0° has been reported. B. Experimental protocol. Upper panel: series of reaching (black dots) and pointing (aftereffect, AF; red dots) trials performed by a representative participant during the entire experimental session. The y axis represents the angular error associated with each movement. Reaching and pointing trials were grouped into blocks delimited by dashed vertical lines. Each block contains 20 reaching and 12 pointing trials, i.e. 32 movements. Note that, for sake of simplicity, only the reaching trials are numbered on the *x* axis. Each block was executed under different lens conditions as indicated by labels above each block (Sham and Prisms conditions). Lower panel: schematic representation of the trial structure. In particualar, the x axis represents the temporal sequence of events (in seconds, s) within a trial, and the y axis the sequence of trials under different lens conditions (labels on the left represent the number of reaching trials; labels on the right indicate the different lens conditions under which the movements were executed). C. Trajectory of a typical reaching movement executed under visual distortion induced by 10° prism lenses (at the -20° target). The dashed black line represents the hand movement trajectory; the dotted blue line represents the projection of mask limits on the movement plane, as empirically determined by fitting a polynomial curve across the points (indicated by asterisks) where the participant starts to see the tip of his right finger. The onset of curvature was identified as the point on the trajectory path where the distance between the real hand trajectory and the ideal trajectory (dashed red line) reached its maximum (continuous red line). D. Profile of tangential velocity of the same movement illustrated in C. The red arrow indicate a clear indentation on the velocity profile due to a corrective sub-movement. In both bottom panels the coloured dots indicate the position (C) of the right finger tip as well as its tangential velocity (D) at the selected time points, as reported in panel D. The target position in C is indicated by the light blue dot.

The same experimental apparatus (see Fig 1A) allowed the participant to perform reaching and pointing trials (i.e. aftereffect trials) according to the procedure described below. During reaching trials, participants were seated at a table containing 4 targets to be reached. The targets (light emitting diode, LED, 5 mm diameter, 2 cd/mq luminance) laid on a circle arc with radius 35 cm centred on the origin of the movement. The movement origin was located 5 cm in front of the subject sternum and aligned with his midline (0°). The four targets were oriented at 0° (i.e., along the mid-sagittal axis), at -40° and -20° (i.e., to the left) and at +20° (i.e., to the right) with respect to the midline. Protruding from each LED, a vertical 1 cm long flag signalled the stop position for each reaching target. A flat cylinder (1 cm diameter) localized on the table on the origin, signalled the movement starting position where the participant had to put the tip of his right index finger. Participants wore a splint on their right arm that fixed the index finger in extension and the other fingers in flexion. The table was provided with a head restraining apparatus, which allowed the reduction of movement artefacts on the EEG and contained a partially occluding semicircular mask that allowed vision of the hand only in the last 40% portion (i.e., approximately the last 14 cm) of the trajectory.

During pointing trials which were executed for evaluation of prism-induced aftereffect, the partially occluding mask was replaced by an occluding mask that totally prevented the vision of the hand and the table. Participants could see only an out-of-reach vertical bar emerging above the mask at a 60 cm distance from the sternum and aligned with their midline.

### Experimental design

The experimental session was organized in blocks of movements each containing series of reaching and pointing (i.e., aftereffect, AF) trials as specified in the following paragraphs.

#### Reaching trials

During reaching trials participants reached the targets under a variable degree of visual shift, that is 0° or 5°, 10°, 15° rightward shifts induced by sham or prismatic lenses, respectively (Fig 1A). In detail (Fig 1B), each reaching trial started at Time 0 when participants were asked to look at a fixation point, i.e. a black dot (1 cm diameter), located on the table 40 cm in front of them along their mid-sagittal axis. Then, at time 3 s, when a target LED was switched on, they were required to look at it, until, at time 5 s, a click provided the GO signal for a reaching movement that should bring their right index finger on the lighted LED flag. Participants had 4 s to complete the movement before the LED switched off. During this time, they were encouraged to correct the trajectory whenever a mismatch was detected between the direction of the hand and the position of the target LED. Once the flag on the lighted LED was reached, they waited till the LED switched off before returning to the starting position.

#### Pointing trials

For evaluation of prism-induced aftereffect (AF) participants were instructed to perform under sham lenses pointing movements towards an out-of-reach midline vertical bar (0,5 cm width) with their right arm laying on the table till its complete extension. No visual feedback was available along the entire hand trajectory. Each AF trial started at Time 0 when participants were asked to look at the vertical bar that appeared over the mask, then, at time 5 s, a click provided the GO signal for the pointing movement. Once having completed the pointing, participants waited for an instruction from the experimenter before returning to the starting position.

#### Block structure

Reaching and pointing trials were grouped into blocks with each block containing 20 reaching and 12 AF trials (total: 32 trials). In particular, three groups of four consecutive AF pointing movements each were inserted after the 4^th^, the 16^th^ and the 28^th^ reaching trial (i.e., trials 5–8, 17–20, and 29–32 were the AF trials). For each participant, data belonging to a group of four AF trials were averaged to obtain a single value. The order of target presentation for reaching trials varied pseudo-randomly fulfilling the criteria that, within each block, each target direction was presented five times and the same direction was never presented consecutively. Each participant performed seven consecutive blocks of 32 movements differing for the type of lens worn according to the following order (Fig 1B): sham (0° visual shift; i.e., baseline), 5° prism, sham (i.e., recovery 1), 10° prism, sham (i.e., recovery 2), 15° prism, sham (i.e., recovery 3). Any lens change, either at the end of each sequence or within each sequence when shifting from reaching to AF trials and *viceversa*, was executed in the eye-closed condition and without explicit information to the participant.

### Movement acquisition and analysis

Right index movements were monitored by using an infrared optoelectronic system (ELITE, BTS, Milano, Italy) which localizes in space markers located on the participant at the sampling rate of 50 Hz. A marker (0,5 cm diameter) was attached to the tip of the right index finger to monitor the trajectory of the movements. A trigger signal allowed us to synchronize acquisition of kinematic data (oversampled at 256 Hz) and EEG data (original sampling: 256 Hz) with target presentation. For each trial, movement acquisition started at Time 0 when the participant positioned his index finger at the starting position (origin; Fig 1A) and ended at time 9 s ensuring movement monitoring during the first 3 s rest period, during the following 2 s period of motionless attention focused on the lighted target and, finally, during the last 4 s period following the acoustic GO signal (Fig 1B). Trials with movements occurring before the GO signal were discarded. Raw data of x/y coordinates of marker displacement and velocity were exported and analyzed offline by custom programs running under MATLAB (The Mathworks, Natwick, USA). In particular, in addition to marker trajectory (Fig 1C) and tangential velocity (TgVel; Fig 1D), for each trial the following parameters were calculated. (a) *Angular error*. For reaching movements, the angular error was taken as the angular difference between two intersecting segments. The former, which represented the ideal correct direction required to reach the target, was drawn between the position where the index finger became visible (i.e., crossed the projection of the mask outline on the table) and the target position; the latter, which represented the actual direction of the movement, was drawn between the position where the index finger became visible and the position where it was 78 ms after the time of the visual feedback appearance. For AF pointing movements, angular error was taken as the angular difference between the segment connecting the origin and the target (i.e., the midline vertical bar) and that connecting the origin and the end positions of the index finger. (b) *Start/end time of movement*. On the basis of a threshold value set at 3% of the peak of TgVel, each movement was provided with both start and end times, taken as the first time after the GO signal when TgVel exceeded and fell below the threshold value, respectively. (c) *Reaction time (RT)*, i.e. the time between GO signal and the movement start. (d) *Movement duration (MT)*, i.e. the time between start and end movement. (e) *Asymmetry index (AI)*, taken as the ratio of the acceleration to the deceleration time. (f) *Linearity index (LI)*, taken as the ratio of the real to the ideal straight movement trajectory length.

### Definition of kinematic anchor points and error estimates for EEG analysis

Time of visual feedback appearance of the hand and time of onset correction were the two anchor points that were used in the subsequent EEG time-frequency analysis.

#### Definition of time of visual feedback appearance

To estimate time of hand visual feedback appearance we first imposed the condition that all participants received a feedback when they had covered 60% of the trajectory. To guarantee that for all participants vision was restricted to the same percentage of trajectory, the distance between the table and the chin rest remained constant (25 cm) while for each participant the partially occluding mask was selected among a series of slightly different semicircular masks (0,5 cm radius difference) to calibrate the mask size to the individual head morphology (Fig 1A). The workspace area beyond which the tip of the right finger became visible was determined empirically for each participant by asking him to perform 5 series of straight slow movements along 9 regularly spaced directions covering the directions from– 50° to 30° and to report the points of index finger appearance (asterisks in Fig 1C). Wearing the splint, which prevented wrist and index movements, and asking the participants to perform the movement with their arm laying on the table, ensured stability of this measure. Such procedure allowed us, first, to identify for each participant the mask size that restricted hand vision to the last 40% of the trajectory, second, to fit across the signed points a curve (polynomial fit) that represented the limits of the occlusion area (pointed curve in Fig 1C). For each reaching trial, the time when movement trajectory crossed this curve was taken as the time of appearance of hand visual feedback (magenta dot on the curve in Fig 1C).

#### Definition of time of onset of movement correction

For trials that required a corrective sub-movement after visual feedback appeared, the time when correction began, i.e. the time of onset of the curvature on the trajectory path, was found as the time when the distance between the marker location and the segment connecting starting and ending positions reached its maximum (continuous red line in Fig 1C).

#### Partition of EEG epochs on the basis of kinematic parameters

For each participant, the median value of the absolute angular error of all reaching trials was used to partition the trials into two groups: high-error, whose angular error was equal or above the median value, and low-error, whose angular error was below the median value.

### EEG acquisition

Continuous EEG activity was acquired from 25 channels by using passive gold EEG electrodes screwed in a preset EEG cap (g.tec Medical Engineering GmbH, Graz, Austria), embedded in a conductive paste (Ten20, Weaver, Colorado, USA), and placed according to the International 10–20 System plus FC3, FC4, CP3, CP4, PO3 and PO4 additional positions. Bipolar electromyographic (EMG) activity from *trapezius* and *pectoralis major* muscles of both sides and ECG activity were recorded by paired disposable surface electrodes (3M Poland, Poland). Bipolar electrooculographic activity (EOG) was recorded by paired surface Au electrodes (g.tec Medical Engineering GmbH, Graz, Austria) placed at the top of the right outer canthus and at the bottom of the left outer canthus to monitor both horizontal and vertical eye movements. EEG, EMG, ECG and EOG activities were filtered (pass band of 0.08–70 Hz), sampled at 256 Hz and digitized at 16 bit (HandyEEG, Micromed, Verona, Italy). Input impedance was kept below 5 kΏ for all electrodes for the entire experimental session. Offline, EEG data were digitally filtered with a band-pass of 2–40 Hz and re-referenced to a common average reference.

### Signal processing

Polygraphic data were analyzed offline using EEGLAB toolbox (Swartz Center for Computational Neuroscience, La Jolla, CA; http://www.sccn.ucsd.edu/eeglab, see [32–34]).

First, after visual inspection of individual traces, we rejected artefacts either related to gross participant displacement or to other sporadically occurring sources (for example: cable movements): this allowed us to exclude that "non-stereotyped" noise influenced the following independent components analysis (ICA) decomposition. In particular, after this cleaning process, about 90% (mean ± S.D.: 88.5±8.4%, n = 12 participants) of the reaching trials has been kept for further analyses. Second, we additionally pruned the data by means of ICA. Maximally independent EEG processes were obtained using FastICA algorithm [35] from the EEGLAB toolbox. On the basis of their scalp map, spectrum and activity, some independent components (ICs) were identified as accounting for blinks, saccades, ECG and muscle artefacts and were used to prune channels EEG data. Third, from the continuous EEG data we extracted epochs of 9 s synchronized with the beginning of the kinematic acquisition of each trial. This allowed us, for each trial, to import on the corresponding EEG epoch the following salient event times: light on, click on, start of the movement, appearance of the hand visual feedback, onset of the movement correction and end of the movement. For each participant, EEG epochs were partitioned and concatenated into two datasets, high- and low-error, according to the angular error of their corresponding movements on a median split basis. Next, 1) both high- and low-error EEG datasets were re-epoched in 3.5 s time windows (from -1.0 to +2.5 s) time-locked to the hand visual feedback appearance, 2) high-error EEG datasets were additionally re-epoched in 3.0 s time windows (from -1.0 to +2.0 s) time-locked to the onset of movement correction. Finally, we performed spectral analysis as detailed below.

#### Time-frequency spectral analysis

To assess event-related spectral amplitude perturbation (ERSP) we performed a time-frequency analysis. In particular, we calculated ERSP, first, by computing the power spectrum over a sliding latency window for each trial, and, then, by averaging across data trials. To compute the spectral estimate, 128 data point moving windows were zero-padded to 1024 points and submitted to a short-time Fourier transform by using a Hanning tapered window yielding an estimate of the power spectrum density with a frequency resolution of 0.25 Hz. Moving windows were centered at 200 evenly spaced latencies from -0.750 s to 2.250 s and from -0.750 to 1.750 s for epochs time-locked to visual feedback appearance and movement correction, respectively.

A finer evaluation of temporal evolution of theta spectral power was obtained by using individual band limits. First, according to Klimesch et al. [36], we defined for each participant his individual alpha frequency (IAF) taken as the frequency bin having, during the resting state, the maximum power density in the range from 6 to 14 Hz averaged over all electrodes. IAF peak was calculated by a standard FFT procedure implemented by a home-made script developed under Matlab (Welch’s method, Hanning windowing function, 0.25 Hz frequency resolution). Then, theta band power was individually calculated as the average of the power obtained for the discrete frequencies within the frequency band from IAF minus 6 Hz to IAF minus 4 Hz [36].

#### Single trial time-frequency spectral analysis

Single-trial spectral analysis was computed on Fz electrode activity by utilizing EEG epochs time-locked on the appearance of the hand visual feedback: we restricted the analysis to the theta frequency band calculated according to IAF. For the Fz channel of each participant, we collected a 3D matrix of data (frequency*times*trials). From that matrix we extracted for each trial the time course of power values (ERSPs, expressed in decibel) within the theta frequency band by averaging between theta frequency bins. Single-trial ERSPs were baseline-corrected by subtracting mean single-trial spectral estimates. To quantify the relationship between single-trial EEG theta activity and error, we first sorted the trials into twenty quantiles of equal size on the basis of the associated angular error. Then, we computed the mean ERSP by utilizing EEG epochs within each quantile and finally we grand-averaged the resulting ERSPs across the 12 participants for each error range.

### Statistical Analysis

Kinematic data were analyzed by using one way or two way repeated measures analysis of variance (one-way or two-way RM ANOVA). Each statistical design is detailed in the Results section. Greenhouse-Geisser correction was used to correct for violation of sphericity when required. Post hoc comparisons were performed by means of Newman-Keuls (N-K) test.

EEG time-frequency plots time-locked to either the hand visual feedback appearance or the onset of movement correction and related statistics were performed using Matlab and EEGLAB toolbox. Power changes occurring after the time-locking event (either visual feedback appearance or onset of movement correction) were assessed at each time-frequency bin by statistical comparisons with respect to the pre-event period (baseline). When low- vs high-error conditions were compared the statistical difference between each corresponding time-frequency bin was computed. In both comparisons (i.e., post- vs pre-event and low- vs high-error), p-values were calculated at each time-frequency point by using a non-parametric permutation statistical method (Wilcoxon test). Bonferroni correction was applied to correct for multiple comparisons and compensate for the fact that a statistical test was performed at each time-frequency point [37]. The same statistical procedure (i.e., Wilcoxon test with Bonferroni correction) was also used to evaluate time frequency plots obtained from data of the single trial analysis. In this case, the power changes occurring after the time-locking event (visual feedback appearance) were assessed at each time-frequency bin with respect to the pre-event period (baseline).

Differences in the temporal evolution of theta frequency scalp maps were assessed by comparing theta power values at each scalp location with paired t test. Bonferroni correction was applied to correct for multiple comparisons [37].

The temporal evolution of the theta power at Fz, calculated on the basis of the IAF, were evaluated on data (extracted from the time-frequency analysis) time-locked either to the visual feedback appearance or to the onset of movement correction. These data were averaged into non overlapping bins of 50 ms, as specified in the Results section. For data time-locked to visual feedback appearance, we applied the Generalized linear mixed model regression for repeated measures by means of a bin*conditions interaction [38] in order to compare, bin-to-bin, the low- and high-error conditions. For data time-locked to the onset of movement correction in the high error condition, we applied the Generalized linear mixed model regression for repeated measures with the time (bin) factor only in order to evaluate the temporal evolution of theta power.

Effect size relative to the paired t-tests was estimated by using the appropriate Cohen’s effects size index (d), while for estimating the effect size relative to the RM ANOVA tests we reported the partial Eta-Squared (η^2^_p_).

Power (1-β) of all the performed tests were calculated and reported. When the power was below the threshold of 0.80 (with α = 0.050) the sample size required to putatively achieve the threshold was reported.

## Results

### Behaviour

#### Baseline: reaching trials

**Fig 2:**
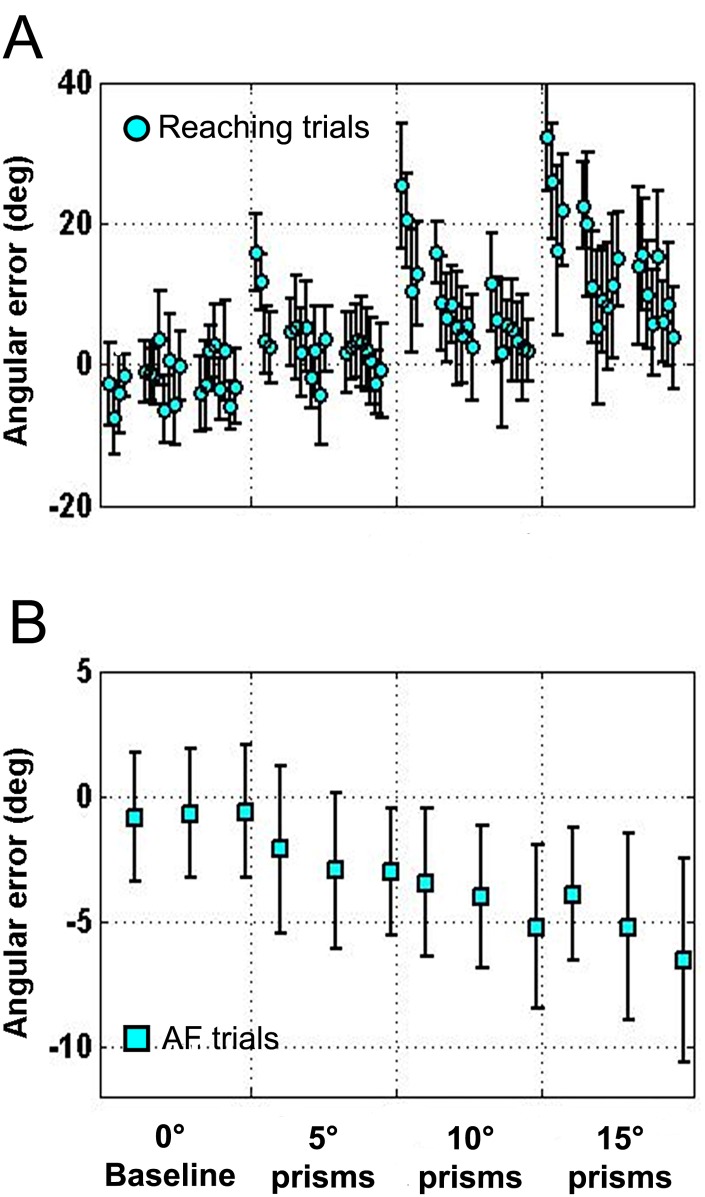
Evolution of the angular error of reaching (A) and pointing (B) movements in subsequent trials performed during baseline and prism blocks. A. Reaching error (light blue dots: mean deg ± SD) at the time when visual feedback appears. Each dot represents the mean value of all participants (n = 12). Each participant performed subsequent reaching movements organized in blocks of 20 movements for each of the following conditions: baseline (0°), 5°, 10°, and 15° prisms exposure. Blocks are indicated by the labels on the x axis of the bottom graph (B). Note the stability of the performance during baseline block, the dependence of the error magnitude on the degree of prism deviation (15° > 10° > 5°) at the early prism exposure, and the progressive error reduction with the repetition of reaching movements. B. Pointing error (aftereffect, AF, light blue squares: mean deg ± SD) measured as angular deviation from midline. Each square represent the mean of all participants (n = 12) obtained after having averaged four consecutive pointing trials for each participant. The four pointing trials were inserted, within each block, after the 4^th^, the 12^th^ and the 20^th^ reaching movement, obtaining three groups of pointing trials *per* block (see labels on the x axis). Note the progressive growth of the angular deviation from midline with prisms exposure, which could be considered a mark of adaptation.

Under basal conditions (i.e., at the first application of sham lenses) all participants executed precise single uncorrected movements towards the requested targets. In particular, participants (n = 12) performed reaching movements with a mean small angular error at the time when feedback became available (Fig 2A, Baseline trials). When averaging separately angular errors committed during reaching movements performed toward the 4 different target directions (data not shown), a small leftward angular error was observed in the 0° target direction (mean ± S.D.: -2.8±3.7 deg) as well as in +20° and -20° target directions (mean ± S.D.: -3.0±3.3 deg and -3.8±3.3 deg, respectively), while in the -40° target direction mean angular error was slightly shifted rightwards (mean ± S.D.: 1.3±4.9 deg). A one-way RM ANOVA with Greenhouse-Geisser correction revealed a significant difference within directions (F_(1.89, 20.76)_ = 4.090, p<0.034; η^2^_p_ = 0.271; 1-β = 0.65, n = 20 to achieve 1-β = 0.8), with errors in the -40° target direction differing from those obtained in the other directions (NK post hoc test: -40° vs +20°, p = 0.031; -40° vs 0°, p = 0.016; -40° vs -20°, p = 0.015). Not only this difference should be taken cautiously due to the suboptimal power of the performed test but also, we did not expect that error differences among directions would bias the results of the EEG analysis due to the equal number of trials executed in the different directions for each lens type. Other reaching movements parameters were fairly homogeneous (mean ± S.D.: RT = 428±108 ms, MT = 1228±227 ms, and TgVel peak = 563±150 mm/s) and only slightly curved (mean LI ± S.D.: 1.049±0.050) with a bell-shaped profile of tangential velocity characterized by a slightly longer deceleration than acceleration phase (mean AI ± S.D.: 0.81±0.26). Appearance of visual hand feedback did not alter the trajectory profile nor cause indentation on the velocity trace, so that, taken together, kinematic data indicate that movements were executed skilfully without corrections regardless of the target direction and occurrence of feedback.

#### Baseline: pointing (i.e., aftereffect) trials

To obtain a reference measure for AF development during the following prism blocks, at the beginning of the experimental session, simple pointing movements under sham lenses were transiently intermingled with basal reaching trials. Four consecutive AF trials were inserted after the 4^th^, the 12^th^ and the 20^th^ reaching movement, obtaining three groups of pointing trials per block. In the case when pointing trials (hereafter called AF) were executed in these basal conditions, i.e. prior to any prism exposure, they showed a minimal mean leftwards deviation from midline (mean ± S.D.: -0.8±2.1 deg), being >1° in only 1 participant and ranging from -1° to +1° in 6 out of 12 participants. Moreover, as expected, all three AF groups remained stable around the participant midline. This led us to exclude an AF development along this baseline block (Fig 2B).

#### Prisms: reaching trials

As expected, participants wearing prisms performed reaching movements with a rightward error at the time of visual feedback appearance whose amount depended on the degree of prism deviation (mean ± S.D., for 5°, 10° and 15°: 3.0±2.2 deg, 8.0±3.2 deg and 13.8±4.8 deg, respectively). A one-way RM ANOVA with Greenhouse-Geisser correction revealed a significant difference in errors within distortions (F_(1.53, 16.80)_ = 104.368, p<0.001; η^2^_p_ = 0.905; 1-β = 1.00). Post hoc NK test revealed a significant difference of mean error for all prism conditions as compared to baseline (0° vs 5°, 10°, 15°; p<0.001) as well as for each prism exposure as compared to the others (5° vs 10°, 15° and 10° vs 15°; p<0.001). Such error was maximum at the beginning of each prism exposure and rapidly decreased within a few trials (Fig 2A). However, due to our experimental protocol, which was characterized by a relatively short exposure to prismatic lens, error remained above baseline level even at the end of prism exposure (Fig 2A). It is worth mentioning that in the recovery phases, when prism lenses where replaced by sham lenses, participants executed leftward errors (i.e., in the opposite direction with respect to prism conditions) that rapidly vanished in the first few trials (Fig 1B, upper panel).

Kinematic analysis showed that large prisms distortions were associated with high-error trials. These trials were characterized both by a change of direction (Fig 1C), indicating movement correction, and by a clear indentation in the profile of tangential velocity (Fig 1D), indicating deceleration. Moreover, a trend was observed in AI and MT so that the greater the distortion, the slower the movement (mean MT ± S.D., for 0°, 5°, 10° and 15° prisms: 1228±227ms, 1253±203 ms, 1314±131 ms and 1362±91 ms, respectively) and the longer its deceleration phase with respect to its acceleration (mean AI ± S.D., for 0°, 5°, 10° and 15° prisms: 0.81±0.26, 0.75±0.23, 0.70±0.13 and 0.60±0.11, respectively).

In fact, a one-way RM ANOVA with Greenhouse-Geisser correction revealed a significant difference for AI within prism distortions (F_(1.86, 20.45)_ = 5.091, p = 0.018; η^2^_p_ = 0.316; 1-β = 0.74, n = 14 to achieve 1-β = 0.8; post hoc NK test: 0° vs 15°, p = 0.004, and 5° vs 15°, p = 0.024) and only a marginal significance for MT within prism distorsions (F_(2.19, 24.06)_ = 2.987, p = 0.065; η^2^_p_ = 0.214; 1-β = 0.55, n = 19 to achieve 1-β = 0.8, post hoc NK test: 0° vs 15°, p = 0.05). In contrast, one-way RM ANOVA with Greenhouse-Geisser correction showed no significant difference within prism conditions in TgVel peak (mean ± S.D., for 0°, 5°, 10° and 15° prisms: 563±150 mm/s, 541±119 mm/s, 525±83 mm/s, and 540±68 mm/s, respectively; F_(1.91, 20.96)_ = 0.410, p = 0.659; η^2^_p_ = 0.036; 1-β = 0.106, n>100 to achieve 1-β = 0.8) and LI (mean ± S.D., for 0°, 5°, 10° and 15° prisms: 1.05±0.05, 1.04±0.04, 1.05±0.04, and 1.13±0.20, respectively; F_(1.23, 13.54)_ = 1.830, p = 0.200; η^2^_p_ = 0.143; 1-β = 0.261, n = 40 to achieve 1-β = 0.8).

#### Prism: pointing (i.e., aftereffect) trials

In analogy with the baseline block, also during prism blocks, we performed simple pointing movements under sham lenses to look for AF development. As expected, the magnitude of the AF (measured as angular deviation from midline) was greater than that observed in basal (0°) condition and was linked to the magnitude of prism deviation (Fig 2B; mean AF error ± S.D., during 0°, 5°, 10° and 15° prism distortions: -0.8±2.1 deg, -2.6±2.6 deg, -4.3±2.5 deg, and -5.0±3.4 deg, respectively). Moreover as can be seen in Fig 2B, which shows the three groups of AF per block, the AF progressively developed from the first to the third group within each block as the number of reaching trials performed under each prism condition increased (see S1 Table for data). To obtain a statistical estimate of the described effect, we performed a 3*4 two-way RM ANOVA with Greenhouse-Geisser correction with lenses (0°, 5°, 10°, 15°, n = 4 levels) and groups of pointing trials (n = 3 levels) as repeated factors. Such analysis revealed a significant effect both for the factor ‘lenses’ (F_(1.83, 20.13)_ = 11.2274, p<0.001; η^2^_p_ = 0.505; 1-β = 0.998) and for the factor ‘group of pointing trials’ (F_(1.94, 21.34)_ = 7.5768, p = 0.003; η^2^_p_ = 0.408; 1-β = 0.877). Post hoc NK test within factor ‘lenses’ revealed a significant difference of mean AF for all prism conditions as compared to baseline (0° vs 5°, p = 0.028; 0° vs 10° and 15°, p<0.001) as well as for prism 5° vs 15° (p = 0.012), and prism 5° vs 10° (p = 0.050), confirming that the magnitude of the AF was linked to the magnitude of prism deviation. Post hoc NK test within factor ‘group of pointing trials’ confirmed AF progression during repeated exposure to the same prism condition by revealing a significant difference of the second (p = 0.046) and the third (p = 0.002) group of pointing trials with respect to the first one.

### EEG: error-related time-frequency responses at scalp locations during reaching trials

Being interested in studying the temporal evolution of spectral changes related to significant time events in the course of the movement, we computed a time-frequency analysis at single channel location time-locked to the appearance of hand visual feedback or to the onset of the corrective movement. Each single reaching movement contributed an EEG epoch and for each participant EEG epochs were concatenated into two datasets, low- and high-error, according to the error size of the corresponding movements partitioned on a median split basis.

It is worth mentioning that we can reasonably exclude EEG data contamination by movement and eye artefacts since 1) displacement of the head was avoided by the restraining apparatus; 2) in both high- and low-error EEG datasets there was no evidence of saccades on the raw EOG trace in our period of interest, i.e. from the appearance of feedback to the end of movement, and 3) EEG analysis was performed on data pruned by ICA.

#### ERSP time-locked to the appearance of visual feedback of the hand at Fz site

**Fig 3:**
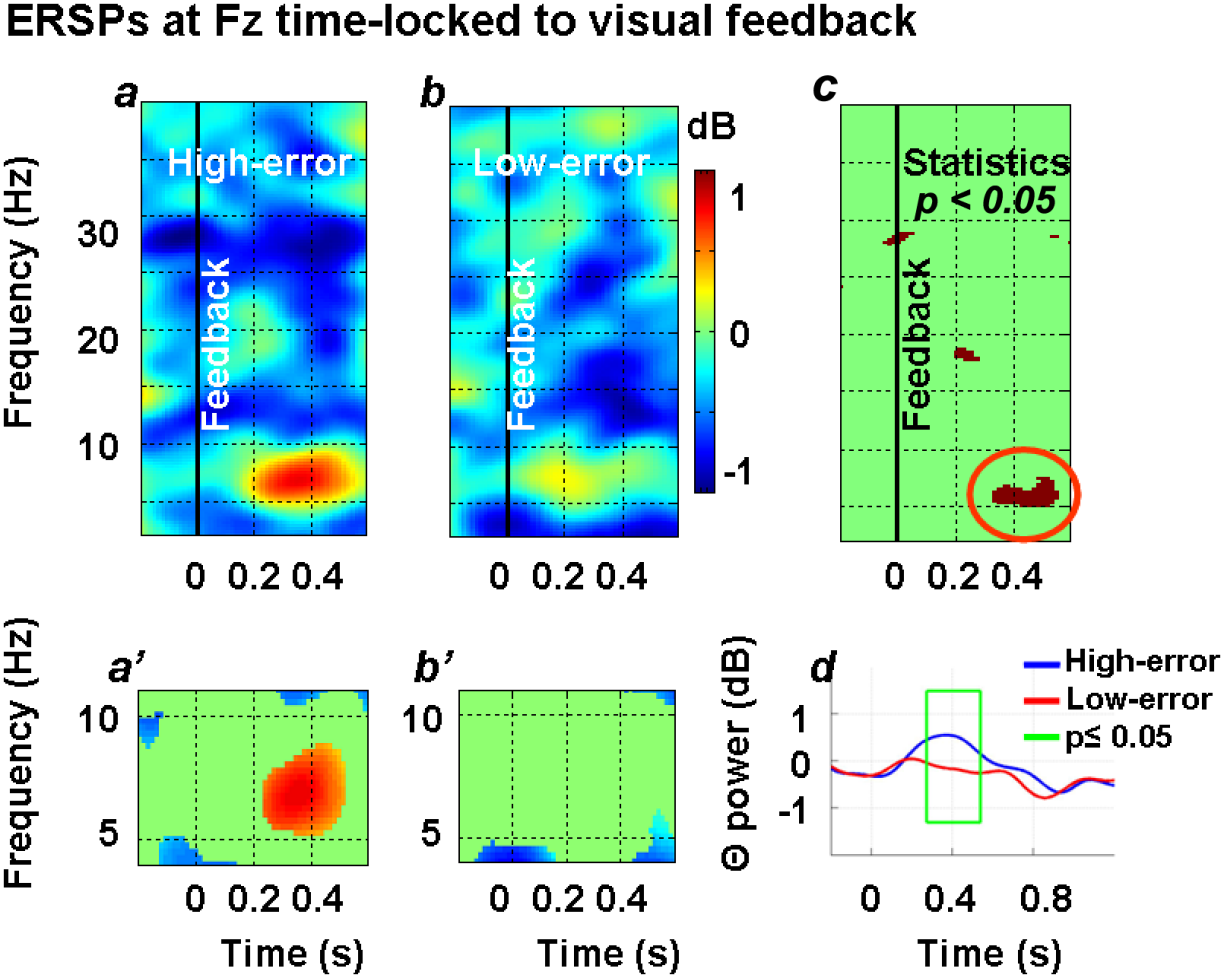
Spatio-temporal dynamics of theta oscillations at Fz channel site time-locked to hand visual feedback appearance in the low- and high-error conditions. Time-frequency representation of the EEG spectral power time-locked to visual feedback appearance (Time = 0 s, black vertical line) in the high- (*a*) and low-error (*b*) conditions. Colour bar indicate power variations in dB. In (*c*) the results of the statistical comparison between the two conditions, (*a*) and (*b*), are reported. Significant time-frequency bins (p-values < 0.05) were coloured in dark red, while others were masked, i.e. replaced, with green. The red ellipse highlights significant values within the theta frequency band. In *a’* and *b’* significant spectral power changes as compared to baseline (Time<0 s) are represented for high- (*a’*) and low-error (*b’*) conditions, respectively. Note that the frequency *window* is restricted in order to highlight changes within the theta frequency band. Non-significant time-frequency power bins are masked with green. In *d*, the time-course of IAF-based theta power at Fz site (averaged over the whole participants population) in the high- (blue) and low-error (red) conditions is represented. The green box shows the time window where the two curves differed maximally.

Fig 3 shows the grand-average (n = 12) time-frequency response time-locked to the appearance of hand visual feedback (Time = 0) in the two conditions of high- (Fig 3a) and low-error (Fig 3b) at Fz electrode site. Starting from approximately 200 ms after feedback appearance and lasting approximately 250 ms, spectral power in the 5–7 Hz frequency range significantly increased with respect to baseline in high-error, but not in low-error related EEG epochs. This result is clearly illustrated in Fig 3a’ and 3b’ that report only the time-frequency bins which resulted statistically different with respect to the baseline period, in the high- and low-error condition, respectively. Moreover, theta power in the high-error condition (Fig 3a) was significantly different as compared to the low-error condition (Fig 3b) as illustrated in the statistical map reported in Fig 3c. In particular, when high- and low-error ERSP data were statistically compared, the difference was significant from 300 to 500 ms after feedback appearance (Fig 3c).

To confirm these results, we further collapsed single frequency bins to compute power analysis within the theta band as a whole. In particular, EEG power computed on Fz ERSPs, filtered and averaged for each participant over theta frequencies (5–7 Hz) and then averaged from 300 to 500 ms, confirmed the effect of error on theta activity. In fact, theta power in the high-error condition (mean ± SD: 0.485 ± 0.553 dB) was significantly higher with respect to the low-error condition (mean ± S.D.: -0.171 ± 0.598 dB) as showed by a paired t-test (t_*(11)*_ = 3.797, p = 0.003; d = 1.09; 1-β = 0.927). A further finer evaluation of the temporal evolution of theta spectral power was obtained by using individual band limits based on IAF (n = 12, mean IAF ± S.D.: 10.8±0.9 Hz, averaged theta limit range from 4.8 to 6.8 Hz). With this aim, we first computed for each participant the individual theta power at each time frame between –250 ms and 1250 ms with respect to hand feedback appearance (Time = 0) in the high- and low-error condition, separately; then, we collapsed these data into 30 non overlapping bins of 50 ms, by averaging the single data points contained in this window. For a robust statistical evaluation of this temporal evolution, we applied to the data a Generalized linear mixed model regression for repeated measures by means of a bin*conditions interaction (1-β = 0.919). The results of this analysis are shown in Table 1 which reports the statistical comparison between the high- and low-error condition at each bin. It appears that theta power was significantly higher in the high-error condition with respect to the low-error condition in two time windows: a) from 250 to 550 ms (p-values comprised between 0.0001 and 0.003), and b) from 750 to 850 ms (p-values comprised between 0.005 and 0.021).

**Table 1:** Evolution of the individual fmθ power segmented in time-windows of 50 ms from -250 to 1250 ms with respect to visual feedback appearance (Time = 0).

Time bin range (ms)	Low error	High error	Comparison H vs L	
	θ power (dB, mean ± SD)	θ power (dB, mean ± SD)	p-value^a^	d-value^b^
-250, -200	-0.044 ± 0.692	-0.091 ± 0.593	0.755	0.09
-200, -150	-0.166 ± 0.702	-0.208 ± 0.460	0.779	0.08
-150, -100	-0.260 ± 0.635	-0.264 ± 0.432	0.976	0.01
-100, -50	-0.302 ± 0.544	-0.276 ± 0.558	0.863	0.05
-50, 0	-0.314 ± 0.583	-0.305 ± 0.815	0.939	0.02
0, 50	-0.272 ± 0.651	-0.327 ± 0.992	0.714	0.11
50, 100	-0.175 ± 0.595	-0.287 ± 0.989	0.452	0.22
100, 150	-0.059 ± 0.446	-0.162 ± 0.909	0.493	0.20
150, 200	0.030 ± 0.281	0.087 ± 0.840	0.665	0.13
200, 250	0.026 ± 0.312	0.309 ± 0.833	0.058	0.55
**250, 300**	-0.042 ± 0.479	0.449 ± 0.808	**0.001**	**0.95**
**300, 350**	-0.115 ± 0.640	0.525 ± 0.722	**<0.0001**	**1.43**
**350, 400**	-0.159 ± 0.694	0.548 ± 0.607	**<0.0001**	**1.37**
**400, 450**	-0.179 ± 0.659	0.512 ± 0.576	**<0.0001**	**1.34**
**450, 500**	-0.229 ± 0.557	0.378 ± 0.626	**<0.0001**	**1.36**
**500, 550**	-0.256 ± 0.416	0.189 ± 0.677	**0.003**	**0.86**
550, 600	-0.240 ± 0.313	0.027 ± 0.703	0.074	0.52
600, 650	-0.216 ± 0.420	-0.082 ± 0.680	0.299	0.30
650, 700	-0.139 ± 0.603	-0.269 ± 0.704	0.383	0.25
700, 750	-0.409 ± 0.920	-0.174 ± 0.545	0.115	0.45
**750, 800**	-0.624 ± 1.097	-0.262 ± 0.491	**0.005**	**0.81**
**800, 850**	-0.761 ± 1.126	-0.417 ± 0.468	**0.021**	**0.66**
850, 900	-0.773 ± 1.001	-0.571 ± 0.480	0.175	0.39
900, 950	-0.674 ± 0.786	-0.660 ± 0.553	0.916	0.03
950, 1000	-0.521 ± 0.556	-0.605 ± 0.667	0.573	0.16
1000, 1050	-0.423 ± 0.459	-0.491 ± 0.722	0.646	0.13
1050, 1100	-0.381 ± 0.491	-0.408 ± 0.729	0.835	0.06
1100, 1150	-0.406 ± 0.522	-0.437 ± 0.733	0.835	0.06
1150, 1200	-0.421 ± 0.528	-0.497 ± 0.768	0.614	0.15
1200, 1250	-0.362 ± 0.610	-0.516 ± 0.800	0.232	0.34

^a^p-value of z-test calculated by means of Generalized linear mixed model regression

^b^Cohen’s effects size index of the z-test.

Fig 3d illustrates the time course of theta power estimated on the basis of the IAF; the green frame highligths the time window (250–550 ms) where the two curves diverge maximally from a statistical standpoint.

#### Single trial analysis of spectral perturbation time-locked to the appearance of visual feedback of the hand at Fz site

**Fig 4:**
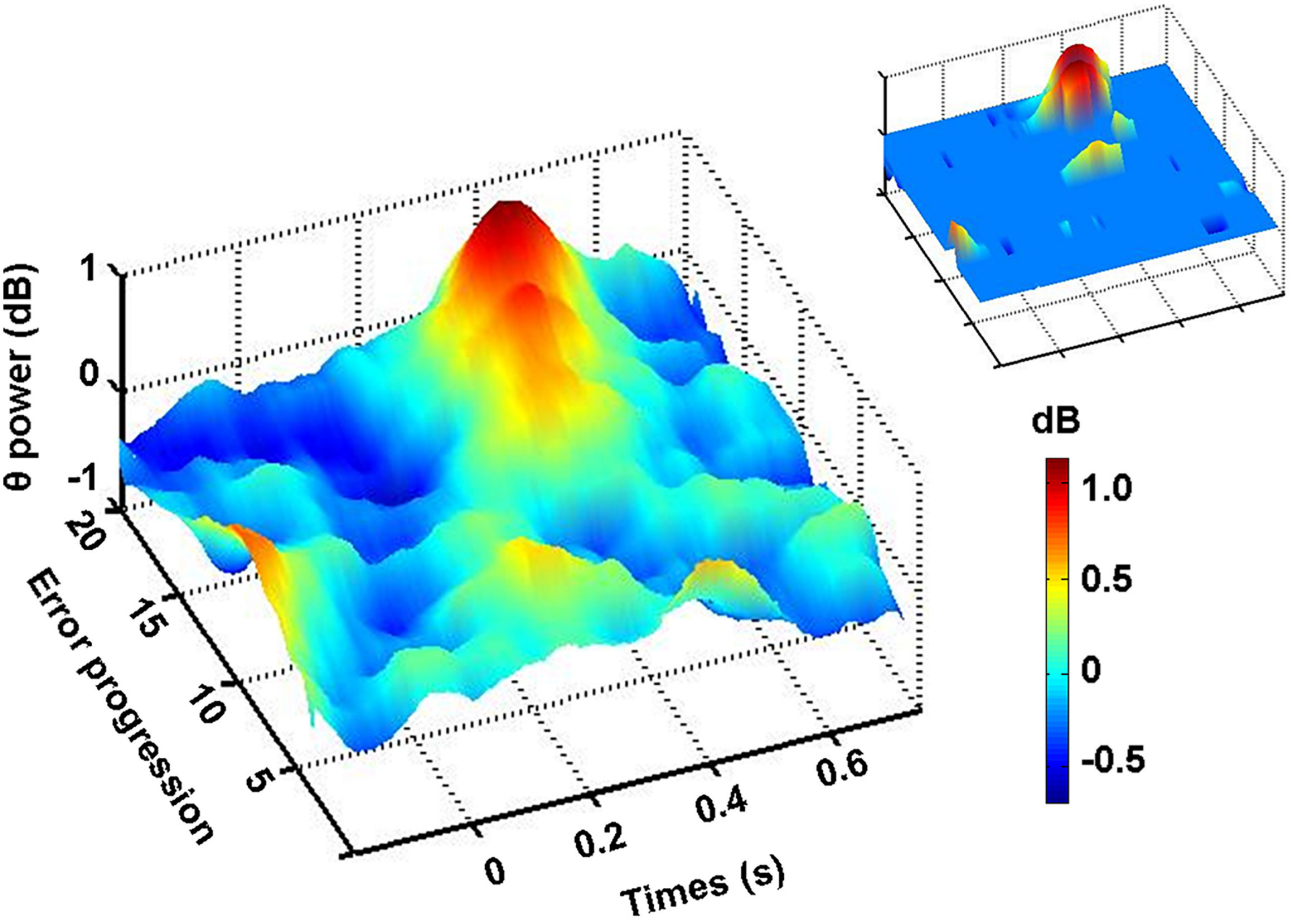
Scaling of IAF-based theta power by the magnitude of motor error. Event-related spectral perturbation (ERSP) in the theta band at Fz channel time-locked to visual feedback appearance (Time = 0 s). Single trials were sorted by the magnitude of error and partitioned in 20 quantiles of increasing errors. In the upper right plot, significant spectral power changes (p< 0.05) as compared to baseline (Time<0 s) are represented. Non-significant time-frequency points (p>0.05) are masked with light blue. x axis: times (s); y axis: error progression; z axis: theta power (dB). Colour bar indicate power variations in dB.

Since a binomial classification of motor error (high versus low) at the time of feedback appearance allowed us to reveal a significant error-related difference of theta activity, we wondered whether a single trial time-frequency analysis could reveal a finer relationship between theta activity and error. Fig 4 plots grand-average ERSP response at Fz site time-locked to feedback appearance. The Fz channel activity was filtered in the theta frequency band according to IAF, smoothed, sorted, and plotted according to the increasing degree of motor error partitioned into twenty quantiles of equal size. As Fig 4 shows, no significant theta modulation with respect to baseline was observed until the 9^th^ quantile was reached. After that, fmθ exhibits a clear significant relation with the size of error peaking at 400 ms following feedback appearance. In the upper right insert of Fig 4, the time frequency bins that reached a statistical significance were reported, while non significant values were masked with light blue.

#### Temporal evolution of theta power scalp maps time-locked to the appearance of the visual feedback of the hand

**Fig 5:**
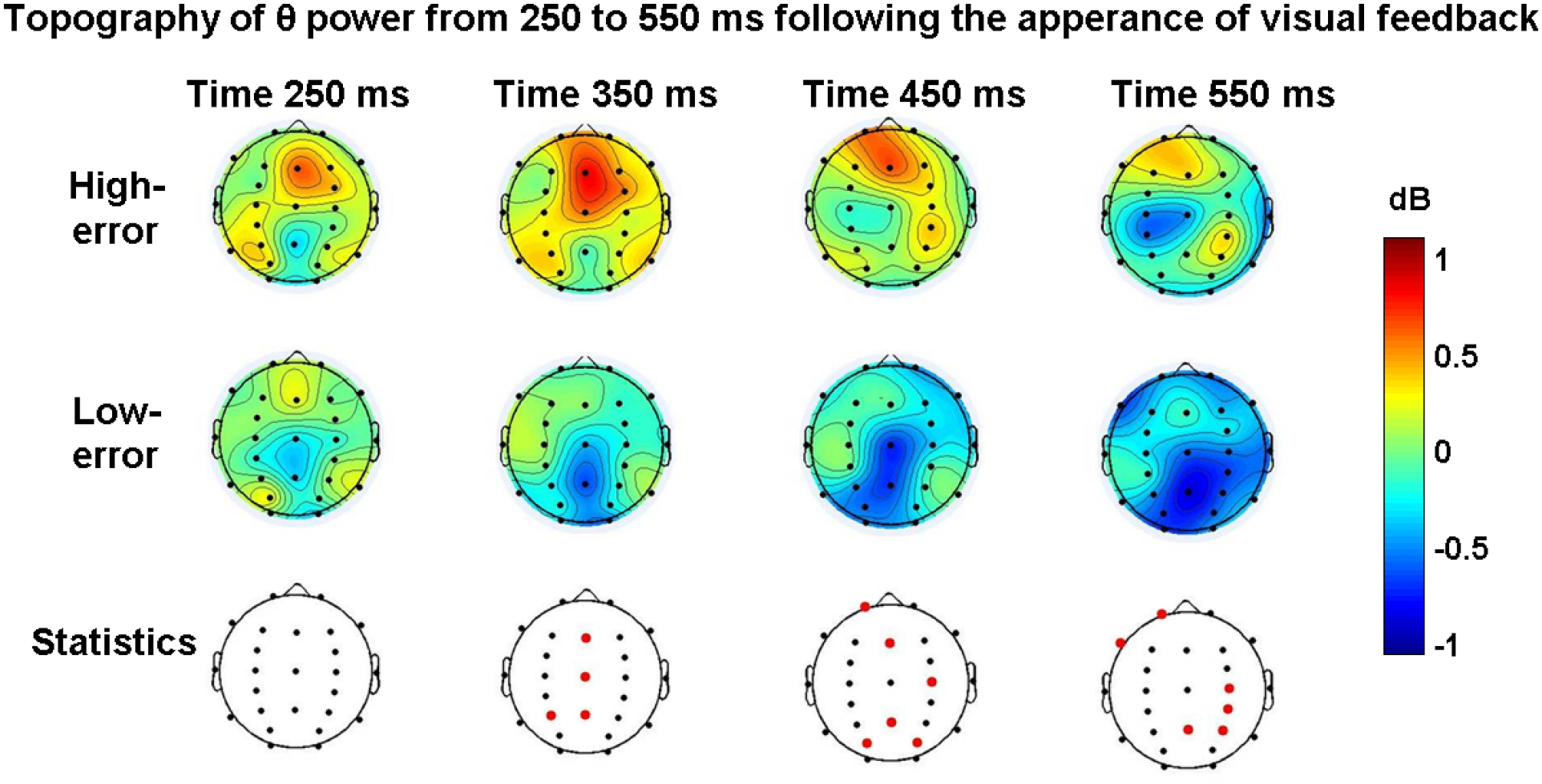
Temporal evolution of theta power scalp maps. Maps were computed at selected times after visual feedback appearance (Time = 0 s) in the high- and low-error conditions (upper and middle rows, respectively). The corresponding statistical comparison between high- and low-error conditions is shown in the lower row: red dots indicate channel locations where the comparison reached statistical significance (paired t test with Bonferroni correction, p≤0.0125). Colour bar indicate power variations in dB.

By analysing ERPSs obtained from other scalp locations, it appeared that, beyond Fz, the difference in theta power in high- versus low-error condition reached a significant level at other electrode sites, such as frontal, central and parietal sites; Fig 5 (upper and middle row) illustrates the temporal evolution of the ERSP maps filtered in theta frequencies and computed at various latencies with respect to feedback appearance in the high- and low-error conditions, respectively. The electrode sites where the statistical comparison between the high- and low-error condition reached a significant value are plotted as red dots in the lower row of Fig 5 (paired t–test with Bonferroni correction to compensate for the fact that a test was performed at each time window; corrected threshold set at p≤ 0.0125; 1-β≥ 0.8 at all significant sites; see S2 Table for the analytical statistical report). The figure highlights that, although theta increase is mostly focused around Fz, theta power is also more represented on parietal sites in the high- vs. low-error condition.

#### ERSP time-locked to the beginning of the corrective movement at Fz site

**Fig 6:**
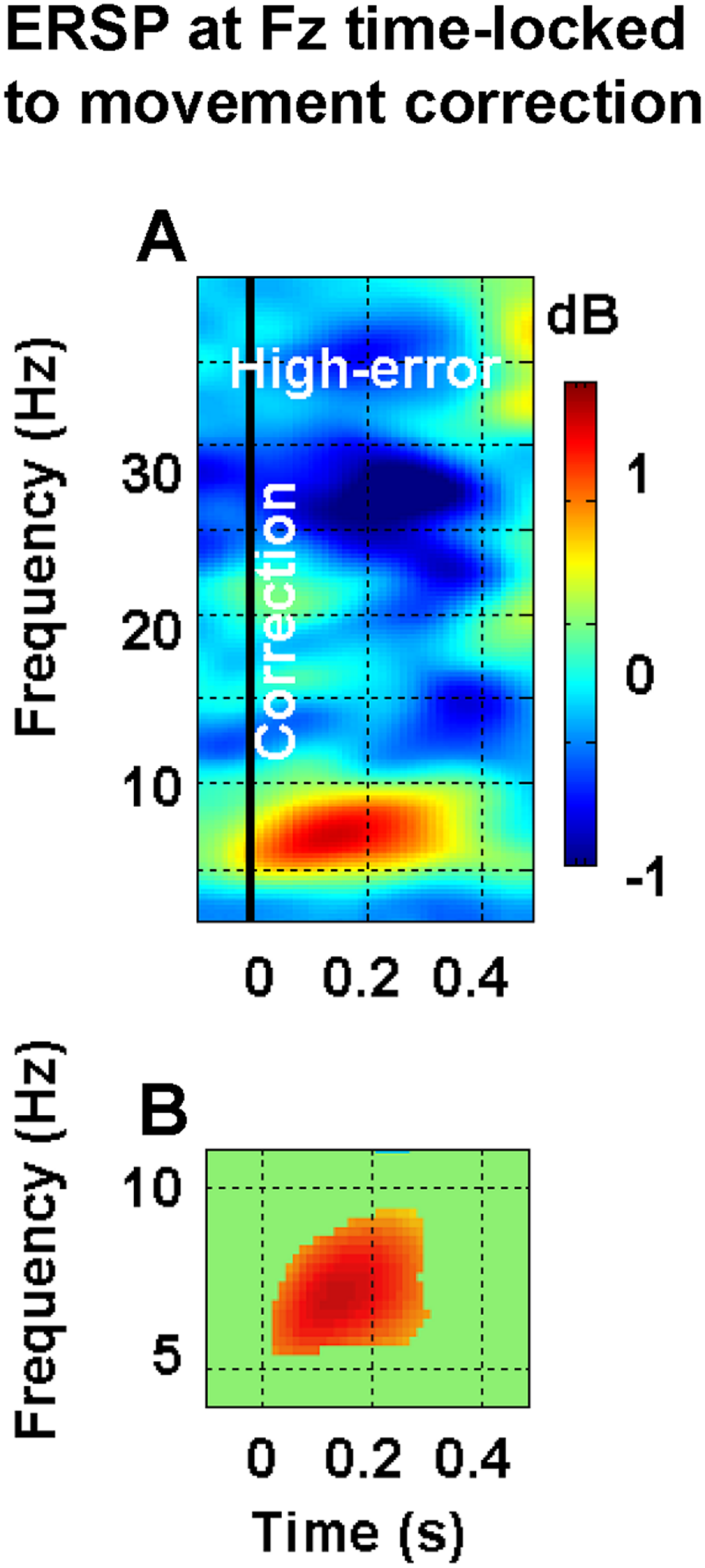
Spatio-temporal dynamics of theta oscillations at Fz channel site time-locked to the onset of movement correction in the high-error condition. A. Time-frequency representation of EEG spectral power time-locked to the onset of movement correction (Time = 0 s, black vertical line) in the high-error condition. B. The same time-frequency representation as in A, but focused on the theta range. Unlike A, only the spectral changes which appeared to be statistically significant as compared to baseline (i.e., Time < 0) were plotted, while non significant time frequency points were masked, i.e. replaced by green. Colour bar indicate power variations in dB.

**Fig 7:**
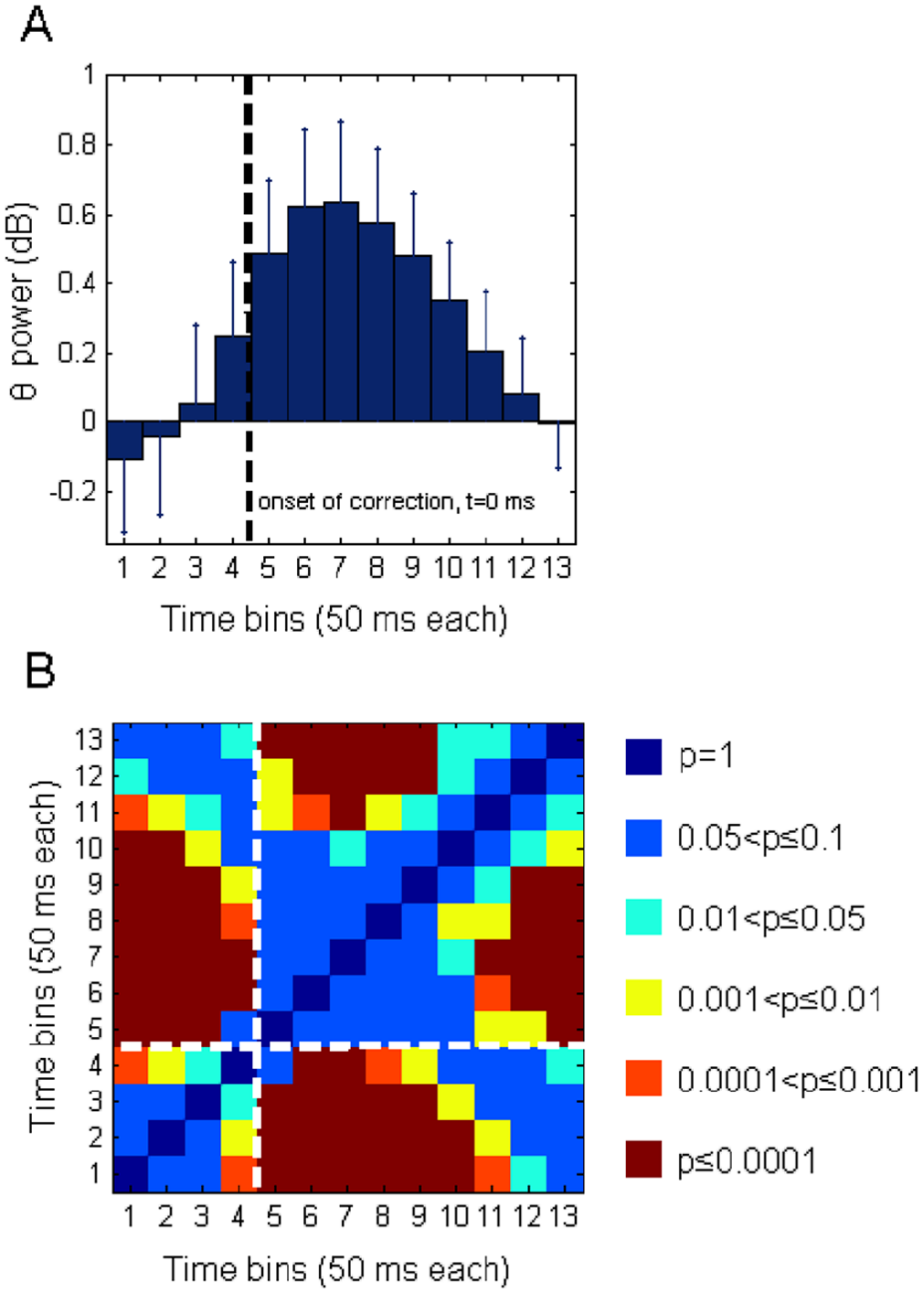
Evolution of theta power in the high-error condition at Fz channel site with respect to the onset of movement correction. A. Evolution of theta power (in dB) from –200 ms to 450 ms, with respect to the beginning of the corrective movement (Time = 0 s, dashed black vertical line). Data were obtained after slicing this time-range into 13 bins of 50 ms each. Bins to the left and to the right of the dashed line precede and follow Time 0, respectively. Error bars represent the standard error of mean. B. Colour matrix of the p-values obtained from the Generalized linear mixed model regression statistics. Columns and rows numbers of the matrix correspond to the time bins of panel A. Each pixel of the matrix reports the corresponding colour-coded p-value obtained from each bin-to-bin comparison (13 x 13). Six non overlapping ranges of p-values were colour coded as indicated on the right. Dashed white lines mark the time bins which precede and follow, respectively, the beginning of the corrective movement (Time = 0 s).

ERSP time-locked to the beginning of the corrective movement were computed for the high-error data set only. In fact, the movements of this data set showed a reliable index of the movement correction onset, since the movements were initially linear and, then, contrary to the low-error trials, started to curve at an easily identifiable time point. As illustrated in Fig 6, when high-error related EEG activity was time-locked to the onset of the corrective movement, theta power increase synchronized with this kinematic trigger (Fig 6A), increasing significantly from approximately 0 to 300 ms with respect to baseline (Fig 6B for statistics). Even in this case, a finer evaluation of the temporal evolution of theta power changes was obtained by utilizing individual theta band limits. With this in mind, we first computed for each participant the individual theta power at each time frame between –200 ms and 450 ms with respect to the beginning of the corrective movement (Time = 0); then, we collapsed these data into 13 non overlapping bins of 50 ms, by averaging the single data points contained in this window. For a robust statistical evaluation of this temporal evolution, we applied to the data the Generalized linear mixed model regression for repeated measures with the time (bin) factor only (1-β = 0.850). The time course of theta power at Fz derived from this analysis is reported in Fig 7A, while the statistical results are shown in Fig 7B which reports the values of significance of the statistical comparison between each time window and the others (see S3 Table for d-values). From the inspection of both figures, it follows that a) theta power remained stable at the baseline level during the first 3 time windows (i.e., between -200 and -50 ms), which did not statistically differ from each other; b) theta power started to increase in the time window between -50 and 0 ms, reached a maximum (mean ± SEM: 0.633 ± 0.232 dB) at the 7^th^ time bin (i.e., between 100 and 150 ms after movement correction), and, then, started to recover to baseline levels; c) complete recovery was observed in the time windows between 350 and 450 ms, as indicated by the fact that during this time frame theta values did not significantly differ from those included between -200 and -50 ms. In particular, since movements in the high-error trials lasted more than 1 s (mean duration ± S.D.: 1116±110 ms), it appears that fmθ was a transient correction-locked response that was exhausted before the movement had ended.

## Discussion

This research has explored the behaviour of frontal midline theta activity -taken as an index of activity generated in the frontal midline cortices- during actions that require on-line correction of misdirected movements. By asking the participants to perform reaching movements under partial visual occlusion of the arm and in the presence of variable degrees of prism-induced visual displacement of the target, we induced the participants to misdirect their movements thus leading to directional errors of different magnitudes. These directional errors serve as visual feedback for the brain as soon as the hand appears beyond the mask (visual occlusion) and force participants to correct their hand trajectory. Evidence from the literature suggests that this error signal is generated mainly by the discrepancy between the predicted visual consequences of movement command elaborated by a forward model and the actual visual sensory estimates (see [2] for review). It is likely that this prediction error triggers movement correction. However, we cannot exclude definitively that a component of this error signal could likewise be generated either from a direct comparison between the position of the arm and that of the target or from a conflict between different feedback signals, that is the veridical proprioceptive signals and the altered visual feedback. We utilized this experimental setting to test some predictions within our model according to which frontal midline structures are engaged when the error is above the threshold that can be managed by the automatic system of correction. In such a case, the high error signal may act also as an alert system increasing the number of areas involved in the control of movement by recruiting the executive network implicated in attentional control. In particular, we tested if frontal midline theta (putatively originated in the mPMF/ACC) was activated by error exceeding a certain threshold and then, scaled with the magnitude of the error. Finally, we tested whether or not the evolution of theta oscillations were correlated in time with significant events such as the onset of visual feedback or the beginning of the corrective movement by conducting event-related spectral analyses. In particular, we investigated the spectral changes in raw EEG channel signals.

### Kinematic data

Our data confirm that under early prism exposure the shift in the perception of the target position induces the participants to misdirect their movements toward the perceived target position, thus eliciting an error in movement direction which is proportional to the degree of prismatic shift. Such error forces the participant to initiate a corrective movement to hit the target which was highlighted, especially in the high error trials, by a clear change in the trajectory direction and by an indentation in the velocity profile of the movement. All participants exhibited corrective movements and were able to hit the target since the mask covered only 60% of the total trajectory and the movements were executed at a speed that allowed the processing of the visual feedback and completion of the reaching task. Following repeated exposure to prisms a progressive shift in the initial direction of movement was observed with the consequence that, when the hand appears beyond the mask, the discrepancy between the direction of the arm and the direction required to hit the target became progressively smaller (i.e., the directional error decreased).

### Sensitivity of frontal-midline theta to motor error

The main result of this study was that the appearance of medial frontal theta oscillations, measured as power increases, was strongly influenced by the magnitude of the directional motor error in a reaching task.

At least three experimental results contribute to confirm this finding. First, when ERSPs were computed separately for each participant and for EEG epochs associated with two different levels of directional error (i.e., high and low) based on a median split, theta power increase at Fz was significantly higher in high- as compared to low-error condition. Second, fmθ increased significantly with respect to baseline only in the high-error condition. Third, EEG analysis on a single-trial basis revealed that, when trials were sorted on a quantile scale with regard to error magnitude, the amount of theta activity increase with respect to baseline was negligible for trials ranked in the lower quantiles. Then theta activity progressively grew as the error increased, thus suggesting a fine relationship between fmθ development and error, when a certain degree of error is exceeded.

There is evidence from the literature that fmθ is generated by the medial prefrontal cortex [10], so that it is likely that the Fz theta activity which we observed to be modulated by error magnitude reflects activation of these cortices. In that sense our results are consistent with previous electrophysiological and brain-imaging studies employing cognitive tasks and requiring elementary motor responses. These studies have shown that 1) monitoring self-performance, such as detecting an error, correcting it [39] and/or signalling the need for behavioural adjustment, is related to medial prefrontal activity, including the ACC [14,39–41]; 2) neural computations in the ACC and functional coupling of ACC with other cortical/subcortical areas seem to be coordinated by theta oscillations [13,15,19,42]; 3) theta activity, whose source is localized in the ACC, seems to be sensitive to error magnitude, that is stronger theta activity is found with larger errors than with smaller ones [43,44].

Our study has explored the behaviour of theta oscillations in a complex and natural motor task (i.e. reaching) in which inaccurate directional performance produced highly identifiable kinematic errors that varied in magnitude across trials. In particular, this is the first study in which brain responses to motor error have been investigated by time-locking EEG signals with the precise appearance of the error visual feedback. In a previous report [27], we found that in a continuous visuo-motor tracking task theta power scaled with the magnitude of error ranked according to three quantiles reflecting different degrees of performance error. Due to the continuous nature of the task, we were not able to study the precise temporal dynamics of these theta changes and therefore these changes could not be locked to any anchor time point which was endowed with a meaning in the motor error domain, such as the appearance of visual feedback or the beginning of the corrective movement.

In the present study, on the other hand, when we centred our EEG epochs (each of which was associated with a reaching trial) on the appearance of hand visual feedback, a transient increase of error-related spectral power in theta frequencies was observed starting approximately 200 ms after hand visual feedback appearance and centred around Fz (fmθ). Re-epoching activity of Fz channel on the onset of the corrective movement, led changes in theta power to synchronize with this new time anchor point with an approximate 50 ms time lead. The observed power increase lasted about 400 ms. Since in our conditions corrective movements lasted more than 1 s, it appears that the fmθ increase we observed was in temporal coincidence with the very early and middle part of movement correction. However, given our experimental design, we can not draw any firm conclusion as to whether theta oscillations reflect the process of error detection or error correction or both. In fact participants had the opportunity to correct their movement in all trials they performed so the experimental design did not allow for comparison of trials associated with correction or not. Moreover, the amount of correction (i.e. the motor adjustment) required for reaching the target co-varied with the magnitude of the errors observed at the time when feedback became available, so that we could not differentiate between the two conditions.

Our data should be discussed by taking into account the results of error-related ERPs studies recorded during complex motor tasks in which inaccurate movement execution produces kinematic errors that vary in magnitude. The comparability with our results derives from the fact that the error- related ERPs may be part of a family of frontocentral negativities generated by a common neuronal network and reflecting phase/amplitude adjustment of frontal theta rhythm sensitive to mismatch signals [31,45]. In particular, Vocat et al. [30] designed a visuomotor adaptation task in which participants performed ballistic pointing movement toward a visual target displayed on a screen and in which variable pointing errors were induced by means of prisms. Vision of their arm was restricted to the very end of each movement, so that on-line movement correction was not allowed. They found a frontocentral ERP related to the touch of the screen, whose components (peaking at 75 and 186 ms) were parametrically modulated by the magnitude of the errors and resembled the ERN and the Pe, respectively. In a more recent work, Torrecillos et al. [31] studied the EEG activity of individuals performing reaching movements in a force field created by a robotic device. Hand path deviations of different sizes were induced by introducing trials in which the force conditions were unpredictably altered, thus avoiding adaptive error-reduction processes. Online corrections were avoided by instructing participants to make shooting movements. In this setting, an error related potential was found, which scaled by the amount of kinematic deviation. This potential, peaking about 250 ms after movement onset and with its maximum located at FCz, resembled the FRN. Finally, Anguera et al. [29] designed a manual aiming task in which movement correction was instead allowed: by employing two groups of participants that adapted to either relatively small (30°) or large (45°) rotation of the visual feedback, they found that, while waveforms time-locked to the onset of the aiming movement did not distinguish between large and small errors, those time-locked to the onset of the corrective movement conversely did. All these studies converge, together with our results, in revealing a parametric modulation of frontal activities by the magnitude of the motor error. The studies of Vocat et al. [30] and Torrecillos et al. [31], which were performed by avoiding error correction, further suggest that the recruitment of medial prefrontal cortex occurs even in the absence of error correction. According to Torrecillos et al. [31], this recruitment may be a sign of error detection rather than error correction. However, in our opinion, the fact that modulation of frontal activity by error can be seen in the absence of correction does not exclude *per se* that the medial frontal cortex could also take part in the cascade of events which leads to error correction, when this condition is required. In this respect, it is of interest to recall the results of Anguera et al. [29] who hypothesized that ERPs time-locked to the onset of the corrective movement and modulated by error “represent the on-line modification of the erroneous movement”.

In our study, theta activity slightly anticipates and co-evolves with error correction. Bearing in mind i) the fact that two independent processes may occur in parallel, and ii) that temporal relationship does not necessarily implicate a causal relationship, this observation calls for further studies to disentangle the issue of theta and mPFC/ACC involvement in error detection, error correction or both. In this regard, the study of Fiehler et al. [39] is of particular interest. In an fMRI setting, they showed that brain activations isolated in the rostral cingular zone and pre-supplementary motor area correlated both with error detection and error correction. They concluded by suggesting the existence of a common neuronal substrate for error detection and correction.

When comparing our work with the above mentioned studies which employed a visuomotor rotation, we found that we had presented a larger set of visuomotor distortions (i.e. 5°, 10°, 15°). This may have allowed us to explore more finely the behaviour of frontal theta reactivity, (i.e., from small errors to relatively large errors). As compared to the other results, our data (especially those related to single trial analysis) seem to indicate that the behaviour of theta was regulated by a sort of threshold mechanism so that a certain amount of error was required to observe theta activation and, *viceversa*, low magnitude errors did not evoke any theta power increase. However, the theta behaviour does not merely function according to an all-or-nothing behaviour. In fact, as the error magnitude grew above a certain level, theta activity progressively increased with increasing error, thus suggesting a fine relationship between fmθ development and error, in the case that an error threshold is exceeded. We may advance the hypothesis that the behaviour of theta activity is related to the degree of the mismatch between the actual and the expected motor outcome, the latter being generated by an internal model within the brain predicting the sensory consequences of movement on the basis of the current state of the arm and the efferent copy of the motor command. Essentially, we interpret theta increase in the high-error condition as a sign of activation of the frontal executive network which becomes sensitive to the error magnitude when a certain degree of error is exceeded. This interpretation seems to be in line with the requirements of the framework also proposed by M. Jeannerod [46]. They found that both control subjects and prefrontal patients, instructed to draw straight lines toward a target under variable degrees of visuomotor mismatch, were able to deviate their trajectories to reach the goal, even if they were not aware of the visuomotor conflict below an average value of 14° of mismatch. Beyond that degree of deviation, healthy participants were aware of the conflict and succeeded in the task, whilst prefrontal patients remained unaware of the conflict and their performance degraded. It was argued that healthy participants compensate automatically for the deviation they were not aware of, by recruiting a network which stores well learned visuomotor response-maps (see [4] for ref.). However, when mismatch between action and sensory consequences exceeds a certain threshold, so that the automatic compensation becomes impossible, awareness of a conflict emerges and a frontal executive network needs to be further recruited to carry on the task successfully.

#### Theta oscillations at parietal cortices during error commission

When we compared theta power behaviour in relation to error commission, at other electrodes in addition to Fz, a significant difference in power between the high- and low-error conditions was also observed at electrodes close to Pz. Considering that Pz overlies parietal medial cortical areas, theta oscillations around Pz may be tentatively attributed to the precuneus, even if a confirmation would require high density EEG recordings and source estimation. In this respect, it is worth noting that the precuneus is involved in processing spatially guided behaviour and in elaborating the body scheme [47,48]. Consequently, it may be activated in ambiguous situations, when the discrepancy between the predicted and actual sensory consequences of actions modulates the sense of self-agency (see [47,48] for ref.). Moreover, the precuneus is implicated in attentional control, by generating an early attentional signal in order to inhibit the prepotent stimulus-response association and reconfigure the stimulus-response mapping [49–51]. Finally, precuneus activation is observed in error-related processes [52,53] and, in particular, it might reflect conscious aspects of error processing [47,48,54] (see also [55] for ref.).

#### Which could be the neuronal substrate of this error-related frontal and parietal recruitment?

Various models have been proposed to explain the activation of frontal midline structures and the generation of theta activity following error commission (see [14,22] for ref.). We suggest that the cerebellum should be investigated for the following reasons. Activation of the cerebellum was strongly increased during a visuomotor rotation task that caused execution errors [5,56]. Moreover, the cerebellum may store forward models of the arm which can be used to predict the consequences of a motor command and evaluate the mismatch between the predicted and actual outcomes [57]. This mismatch signal may recruit the executive network as suggested by the fact that prediction error generates frontocentral negative potentials whose sources include the medial prefrontal cortex [31]. In addition, we previously observed that, during a tracking task cerebellar patients failed to exhibit an increase of both error-related fmθ and theta coherence between ACC and the precuneus [27]. Moreover, several direct or indirect pathways may convey this cerebellar generated signal to the ACC: there are in fact reciprocal connections among cerebellar, parietal and prefrontal cortices [58,59], including medial-prefrontal cortex [60]. Furthermore, at least one electrophysiological study in humans, utilizing single-pulse transcranial magnetic stimulation (TMS), has shown that the cerebellum can evoke theta activity within the frontal cortex [61].

#### Relationship between frontal-midline theta and indices of adaptation

It is commonly presumed that motor errors correction due to prism visual displacement depends on an early transient “strategic” motor control process followed by a late long-lasting plastic process, which implies spatial realignment of the visual map with the arm proprioceptive map [62–66]. The early process allows for rapid reduction in reaching endpoint errors and is obtained by selecting strategies that minimize error on the basis of the previous movement. This initial stage is accompanied and then followed by adaptation (i.e., learning a new visuomotor map), which is indicated by the development of an aftereffect.

We found that, on average, our population did develop an aftereffect. Since aftereffect is an index of true adaptation, the question may be posed if the theta oscillations we observed in the high-error condition play a role in learning and/or storage of a new visuomotor mapping. This process may require the integrity of the parietal cortex [4]. Of course, adaptation may occur in the absence of frontal activation as when low degrees of visuomotor rotation produce adaptation but not theta oscillations. However, it cannot be excluded that theta associated with high-error commission may represent a strengthening mechanism to boost adaptation in downstream brain regions.

## Conclusions

### A heuristic proposal for future research

The observation that frontal midline theta scaled with the magnitude of error in the high-error condition and did not increase in the low-error condition leads us to advance the following working hypothesis: a) errors of small magnitude, which do not call for the revaluation of the motor strategy by the executive system, are corrected on the fly with the intervention of the usual sensory-motor network, possibly by utilizing a visually-driven error feedback correction; b) errors of large magnitude, which may not be corrected by utilizing the usual sensory-motor scheme, may necessitate more cognitive control by recruiting the frontal midline structures.

From the results of the present study, we attempt to configure the following scenario. Activation of the mPFC/ACC occurs whenever an error signal calls for major performance adjustments. This signal may indeed be generated in the cerebellum, which is known to contain forward models for prediction, by comparing predicted and actual outcome (see also [9]). If this signal exceeds a certain threshold, it leads to recruitment of the mPFC/ACC and the related executive network. The activated mPFC/ACC might then exert a top-down control by selecting the appropriate motor module for motor correction [67]. It will be challenging to investigate if this control also plays a role in boosting learning of a new visuo-motor association.

## Supporting Information

S1 Table Mean pointing movement (AF) errors per each lens condition (block). (DOC)

S2 Table High- vs low-error theta power comparisons at different sites and latencies after visual feedback appearace. (DOC)

S3 Table Cohen’s effect size indexes (d) for z-test corresponding to the analysis represented in Fig 7. (DOC)
