# Supplementary material for: EEG Theta Dynamics within Frontal and Parietal Cortices for Error Processing during Reaching Movements in a Prism Adaptation Study Altering Visuo-Motor Predictive Planning
Source: PLoS One. 2016 Mar 10;11(3):e0150265. doi: 10.1371/journal.pone.0150265 (PMC4786322; doi:10.1371/journal.pone.0150265)
Supplement: S1 Table — (DOC) [file pone.0150265.s001.doc]

S1 Table. Mean pointing movement (AF) errors *per* each lens condition (block).

|  | 1^st^ AF group | 2^nd^ AF group | 3^rd^ AF group |
| --- | --- | --- | --- |
| 0° lens | -0.876 ± 2.376 | -0.790 ± 2.400 | -0.668 ± 2.391 |
| 5° lens | -2.090 ± 2.956 | -2.936 ± 3.032 | -2.835 ± 2.481 |
| 10° lens | -3.475 ± 2.594 | -4.041 ± 2.355 | -5.219 ± 3.015 |
| 15° lens | -3.903 ± 2.511 | -5.066 ± 3.722 | -6.200 ± 4.025 |

1^st^, 2^nd^, and 3^rd^ AF groups: AF (mean deg ± SD) at early, middle, and late exposure.
