# Supplementary material for: EEG Theta Dynamics within Frontal and Parietal Cortices for Error Processing during Reaching Movements in a Prism Adaptation Study Altering Visuo-Motor Predictive Planning
Source: PLoS One. 2016 Mar 10;11(3):e0150265. doi: 10.1371/journal.pone.0150265 (PMC4786322; doi:10.1371/journal.pone.0150265)
Supplement: S2 Table — (DOC) [file pone.0150265.s002.doc]

S2 Table. High- vs low-error theta power comparisons at different sites and latencies after visual feedback appearace.

| **350 ms** | | | **450 ms** | | | **550 ms** | | |
| --- | --- | --- | --- | --- | --- | --- | --- | --- |
| **Fz** | t=3.101 | θ_H_=0.640±0.664 | **Fp1** | t=4.089 | θ_H_=0.499±0.832 | **Fp1** | t=4.168 | θ_H_=0.257±0.656 |
|  | p=0.010 | θ_L_=-0.037±0.719 |  | p=0.002 | θ_L_=-0.319±0.728 |  | p=0.002 | θ_L_=-0.542±0.548 |
|  | d=0.895 |  |  | d=1.180 |  |  | d=1.203 |  |
|  | 1-ß=0.771 |  |  | 1-ß=0.960 |  |  | 1-ß=0.967 |  |
|  | | | | | | | | |
| **Cz** | t=3.503 | θ_H_=0.356±0.992 | **Fz** | t=3.748 | θ_H_=0.500±0.603 | **F7** | t=5.167 | θ_H_=0.091±0.501 |
|  | p=0.005 | θ_L_=-0.433±0.753 |  | p=0.003 | θ_L_=-0.178±0.614 |  | p<0.001 | θ_L_=-0.770±0.492 |
|  | d=1.011 |  |  | d=1.082 |  |  | d=1.492 |  |
|  | 1-ß=0.876 |  |  | 1-ß=0.920 |  |  | 1-ß=0.998 |  |
|  | | | | | | | | |
| **P3** | t=3.302 | θ_H_=0.198±0.477 | **C4** | t=4.402 | θ_H_=0.106±0.736 | **C4** | t=3.410 | θ_H_=-0.110±0.528 |
|  | p=0.007 | θ_L_=-0.372±0.811 |  | p=0.001 | θ_L_=-0.335±0.872 |  | p=0.006 | θ_L_=-0.576±0.546 |
|  | d=0.953 |  |  | d=1.271 |  |  | d=0.984 |  |
|  | 1-ß=0.829 |  |  | 1-ß=0.981 |  |  | 1-ß=0.855 |  |
|  | | | | | | | | |
| **Pz** | t=3.210 | θ_H_=-0.134±0.486 | **Pz** | t=4.967 | θ_H_=0.005±0.434 | **P4** | t=6.574 | θ_H_=0.134±0.456 |
|  | p=0.008 | θ_L_=-0.654±0.548 |  | p<0.001 | θ_L_=-0.746±0.406 |  | p<0.001 | θ_L_=-0.794±0.289 |
|  | d=0.927 |  |  | d=1.434 |  |  | d=1.898 |  |
|  | 1-ß=0.803 |  |  | 1-ß=0.996 |  |  | 1-ß=1.000 |  |
|  | | | | | | | | |
|  |  |  | **PO3** | t=3.257 | θ_H_=0.035±0.705 | **CP4** | t=5.161 | θ_H_=0.244±0.514 |
|  |  |  |  | p=0.008 | θ_L_=-0.604±0.753 |  | p<0.001 | θ_L_=-0.806±0.400 |
|  |  |  |  | d=0.940 |  |  | d=1.490 |  |
|  |  |  |  | 1-ß=0.816 |  |  | 1-ß=0.998 |  |
|  | | | | | | | | |
|  |  |  | **PO4** | t=3.235 | θ_H_=0.030±0.369 | **Pz** | t=3.091 | θ_H_=-0.106±0.581 |
|  |  |  |  | p=0.008 | θ_L_=-0.413±0.514 |  | p=0.010 | θ_L_=-0.869±0.507 |
|  |  |  |  | d=0.934 |  |  | d=0.892 |  |
|  |  |  |  | 1-ß=0.811 |  |  | 1-ß=0.768 |  |

θ_H_ = mean theta power (dB) ± SD in the high-error condition; θ_L_ = mean theta power (dB) ± SD in the low-error condition; p = p-value; d = Cohen’s effect size index fot paired t-test; 1-ß = power of the performed test
