# Supplementary material for: EEG Theta Dynamics within Frontal and Parietal Cortices for Error Processing during Reaching Movements in a Prism Adaptation Study Altering Visuo-Motor Predictive Planning
Source: PLoS One. 2016 Mar 10;11(3):e0150265. doi: 10.1371/journal.pone.0150265 (PMC4786322; doi:10.1371/journal.pone.0150265)
Supplement: S3 Table — (DOC) [file pone.0150265.s003.doc]

S3 Table. Cohen’s effect size indexes (d) for z-test corresponding to the analysis represented in Fig. 7.

| Bin n° | **1** | **2** | **3** | **4** | **5** | **6** | **7** | **8** | **9** | **10** | **11** | **12** | **13** |
| --- | --- | --- | --- | --- | --- | --- | --- | --- | --- | --- | --- | --- | --- |
| **1** |  | 0.157 | 0.348 | 0.933 | 1.361 | 1.854 | 1.956 | 1.827 | 1.663 | 1.163 | 0.918 | 0.582 | 0.341 |
| **2** | 0.157 |  | 0.191 | 0.768 | 1.214 | 1.697 | 1.799 | 1.670 | 1.498 | 1.017 | 0.761 | 0.425 | 0.184 |
| **3** | 0.348 | 0.191 |  | 0.568 | 1.036 | 1.506 | 1.608 | 1.480 | 1.296 | 0.841 | 0.570 | 0.234 | 0.007 |
| **4** | 0.933 | 0.768 | 0.568 |  | 0.555 | 1.014 | 1.121 | 0.986 | 0.767 | 0.354 | 0.031 | 0.322 | 0.575 |
| **5** | 1.361 | 1.214 | 1.036 | 0.555 |  | 0.369 | 0.464 | 0.344 | 0.116 | 0.177 | 0.505 | 0.818 | 1.043 |
| **6** | 1.854 | 1.697 | 1.506 | 1.014 | 0.369 |  | 0.102 | 0.027 | 0.291 | 0.554 | 0.936 | 1.272 | 1.513 |
| **7** | 1.956 | 1.799 | 1.608 | 1.121 | 0.464 | 0.102 |  | 0.129 | 0.399 | 0.648 | 1.038 | 1.374 | 1.615 |
| **8** | 1.827 | 1.670 | 1.480 | 0.986 | 0.344 | 0.027 | 0.129 |  | 0.263 | 0.529 | 0.909 | 1.246 | 1.486 |
| **9** | 1.663 | 1.498 | 1.296 | 0.767 | 0.116 | 0.291 | 0.399 | 0.263 |  | 0.312 | 0.696 | 1.050 | 1.304 |
| **10** | 1.163 | 1.017 | 0.841 | 0.354 | 0.177 | 0.554 | 0.648 | 0.529 | 0.312 |  | 0.313 | 0.624 | 0.847 |
| **11** | 0.918 | 0.761 | 0.570 | 0.031 | 0.505 | 0.936 | 1.038 | 0.909 | 0.696 | 0.313 |  | 0.336 | 0.577 |
| **12** | 0.582 | 0.425 | 0.234 | 0.322 | 0.818 | 1.272 | 1.374 | 1.246 | 1.050 | 0.624 | 0.336 |  | 0.241 |
| **13** | 0.341 | 0.184 | 0.007 | 0.575 | 1.043 | 1.513 | 1.615 | 1.486 | 1.304 | 0.847 | 0.577 | 0.241 |  |
